# Association between waist triglyceride index, body mass index, dietary inflammatory index, and triglyceride- glucose index with chronic kidney disease: the 1999-2018 cohort study from NHANES

**DOI:** 10.3389/fendo.2024.1390725

**Published:** 2024-08-05

**Authors:** Zhiyu Li, Zongping Xu, Chenhao Xuan, Hongyang Xu

**Affiliations:** Department of Critical Care Medicine, The Affiliated Wuxi People’s Hospital of Nanjing Medical University, Wuxi People’s Hospital, Wuxi Medical Center, Nanjing Medical University, Wuxi, China

**Keywords:** dietary inflammatory index, triglyceride glucose index, waist triglyceride index, body mass index, chronic kidney disease

## Abstract

**Purpose:**

To compare the dietary inflammatory index (DII), triglyceride glucose index (TyG), waist triglyceride index (WTI), and body mass index (BMI) in predicting the survival of chronic kidney disease (CKD).

**Methodology:**

Inclusion of 23,099 participants from the NHANES database who met specific criteria. Baseline was established using quartiles of DII index. The relationship between DII index, WTI index, TyG index, and BMI index with mortality rate in CKD patients was evaluated using Kaplan-Meier curves. Univariate and multivariate COX regression risk models were used to study the relationship between DII index, WTI index, and TyG index with mortality risk in CKD patients. Stratification of eGFR by age and gender was conducted to investigate the association between DII index, WTI index, and TyG index with mortality risk in CKD patients. Restricted cubic spline analysis was used to study the correlation between DII index, WTI index, and TyG index with mortality risk in CKD patients.

**Results:**

The incidence of CKD increased with the increase of DII index, WTI index and TyG index. After multivariable adjustment, the fourth quartile of DII index, TyG index and WTI index showed the highest risk for CKD [DII: hazard ratio (HR) 1.36, 95% confidential interval (CI) (1.23–1.51); TyG: HR 1.21; 95% CI (1.07–1.37); WTI: HR 1.29; 95% CI (1.13–1.46)]. There was no difference in the risk of developing CKD between the obese group (BMI ≥24 kg/m2) and the normal weight group (P>0.05).

**Conclusion:**

This study has identified a significant association between elevated DII index, WTI index, and TyG index with the risk of CKD. Furthermore, the DII index demonstrated superior prognostic capability in predicting CKD compared to other indicators.

## 1 Introduction

The prevalence of chronic kidney disease continues to increase among adults in the United States (1). Chronic kidney disease is characterized by an estimated low glomerular filtration rate (eGFR) or the presence of albuminuria, but is often diagnosed when the bilateral glomerular filtration rate falls to very low levels (eGFR ≤60mL/min/1.73m²) (2). In order to more effectively prevent end-stage uremia resulting from chronic kidney disease (3), dietary adjustments can be a crucial method for preventing the onset of chronic kidney disease (4, 5).

Since cardiovascular diseases, hypertension, diabetes and obesity are all likely to lead to chronic kidney disease (6–9) the corresponding index can be used to predict the occurrence of chronic kidney disease. For example, the TyG index used the combination of fasting glucose and triglycerides as a reliable indicator to assess insulin resistance (IR) (10), and its increase was positively correlated with renal failure in the elderly (11). In addition, TyG index can also effectively predict all-cause mortality related to impaired renal function (12). WTI index consists of the combination of waist circumference and triglycerides, which is associated with metabolic syndrome and acute pancreatitis (13, 14)while WTI index has not been involved in chronic kidney disease studies. A restricted diet is essential for managing chronic kidney disease, and the DII index can be used as a dietary assessment tool that includes a variety of anti-inflammatory and pro-inflammatory diets. Through investigation of adult diabetes, IR and dietary patterns, it has been found that dietary elements that promote inflammation may increase the risk of IR and diabetes (15, 16), and DII may also affect the incidence of cardiovascular disease in Americans and muscle osteoporosis in patients with chronic kidney disease (17). Whether diet has an advantage over other indicators has not been reported.

Therefore, NHAES data were used to explore the relationship between DII index, TyG index, WTI index and BMI index and chronic kidney disease in American adults.

## 2 Methods

### 2.1 Study population

**Figure 1:**
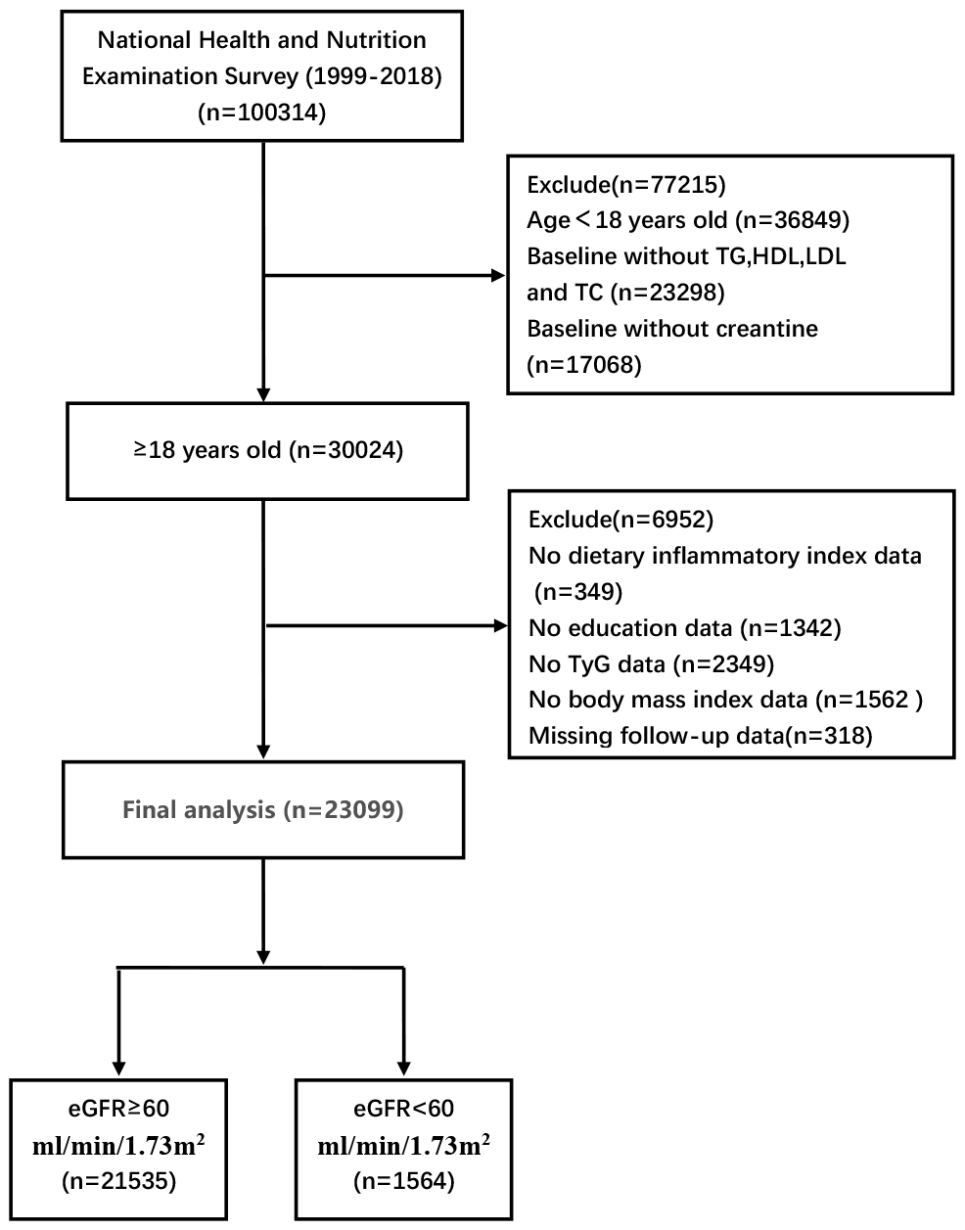
Inclusion and exclusion process of the NHANES 1999–2018.

This retrospective analytic study was conducted among U.S. adults aged 18 years and older in NHANES 1999–2018, a comprehensive nationwide health survey administered by the Centers for Disease Control and Prevention. A total of 100,314 participants were initially enrolled in the study, with specific exclusion criteria including age, fasting lipid levels and missing baseline demographic characteristics. Based on these criteria, 23,099 participants were ultimately included in the analysis (**Figure 1**).

### 2.2 Exposure and outcomes

The baseline assessment includes the collection of biochemical markers from plasma by trained personnel following strict storage and handling protocols. Biochemical markers such as fasting blood glucose (FBG), triglycerides (TG), total cholesterol (TC), serum creatinine, high density lipoprotein cholesterol (HDL-C), low density lipoprotein cholesterol (LDL-C), and total cholesterol (TC) were analyzed (18, 19). Waist circumference, body mass index (BMI), educational attainment, and ethnic categories were obtained through computer-assisted personal interviews (CAPI) conducted at the participants’ homes. The WTI index is calculated using the formula: waist circumference (cm) * triglycerides (mmol/L) (20). The calculation of the DII involves determining an individual’s DII score from 28 diets (21) in order to assess the potential impact of diet on inflammation. The procedure for calculating the DII is as follows: Z-values are calculated based on the mean and standard deviation of the parameters of the 28 food items ((Estimated Intake of an Individual - Global Standardized mean)/(standard deviation)). The Z-value for each food parameter is converted to a percentile value and centered by doubling the value and then subtracting “1”. The centered percentile value for each food parameter is multiplied by the inflammation score for each parameter to arrive at a “food-specific parameter DII score.” Finally, all of the “food-specific parameter DII scores” are added together to calculate the individual’s DII score. Calculation formula for estimating bilateral glomerular filtration rate (eGFR) based on an all-age spectrum correction equation for creatinine in 2021. The primary outcome of this study was to compare the correlation of WTI index, DII index and TyG index with all-cause mortality in patients with chronic kidney disease.

### 2.3 Statistical analysis

To enhance data accuracy and mitigate potential biases arising from NHANES’ intricate multi-stage sampling design, this study employed sample weighting in accordance with NHANES guidelines. Weighted percentages were used for categorical variables while weighted averages and standard errors were utilized for continuous variables. The researchers conducted weighted multiple logistic regression to explore correlations between three indices (TyG index, DII index, and WTI index) and CKD, adjusting for various covariates. Kaplan-Meier survival curves were used to assess the predictive impact of the WTI index, DII index, TyG index, and BMI on all-cause mortality in patients with CKD. Restricted cubic spline plots were drawn to illustrate the associations between the DII index, TyG index, and WTI index and the risk of CKD. Univariate and multivariate Cox regression models were used to analyze the sensitivity of the DII index, WTI index, and TyG index in testing for CKD. Model 1 was unadjusted, Model 2 was adjusted for sex, age, and BMI, and Model 3 included systolic blood pressure (SBP), total cholesterol (TC), age, education, BMI, serum creatinine, smoking habit, sex, and race as factors. The data were analyzed using R language (version 4.2.1) and SPSS 27.0 software with a statistical significance level set at p<0.05.

## 3 Results

### 3.1 Characteristics

The study population consisted of 23,099 participants from the NHANES database who met specific criteria. The DII index showed a fourfold increase and was statistically significant in SBP, BMI, age, total triglycerides (TG), waist circumference, diabetes status, education level, gender, smoking status, FBG, TC, race, and CKD subtypes. Non-Hispanic White had the highest number of baseline characteristics at about 44.2%, followed by Mexican American at about 18.6% (**Table 1**).

**Table 1:** Weighted baseline characteristics of the study subjects, NHANES 1999–2018.

Variable(s)	Dietary Inflammation Index				*p*
	Quartile 1	Quartile 2	Quartile 3	Quartile 4	
N (%)	5804 (25.13)	5754 (24.91)	5770 (24.98)	5771 (24.98)	
SBP (mmHg)	113.91 ± 37.59	112.93 ± 40.07	113.41 ± 40.60	110.38 ± 43.72	<0.001
BMI (kg/m2)	27.98 ± 6.33	28.44 ± 6.58	28.99 ± 7.04	29.19 ± 7.43	<0.001
Serum creatinine (mg/dl)	0.87 ± 0.28	0.87 ± 0.32	0.87 ± 0.35	0.88 ± 0.39	0.151
Age (year)	48.68 ± 17.90	47.54 ± 18.78	47.81 ± 19.03	47.16 ± 19.40	<0.001
HDL-C (mmol/l)	1.40 ± 0.42	1.40 ± 0.41	1.39 ± 0.42	1.38 ± 0.41	0.219
TG (mg/dl)	1.53 ± 1.33	1.55 ± 1.36	1.52 ± 1.46	1.40 ± 1.10	<0.001
TG (mmol/l)	135.81 ± 117.49	136.92 ± 120.12	134.21 ± 129.71	123.83 ± 97.02	<0.001
LDL-C (mmol/l)	2.86 ± 1.06	2.85 ± 1.05	2.87 ± 1.07	2.86 ± 1.06	0.719
Waist (cm)	97.32 ± 15.27	98.01 ± 16.14	98.84 ± 16.44	98.78 ± 16.86	<0.001
Diabetes (%)					<0.001
Yes	548 (9.4)	630 (10.9)	666 (11.5)	688 (11.9)	
No	5256 (90.6)	5124 (89.1)	5104 (88.5)	5083 (88.1)	
Education of level (%)					<0.001
<high school	1376 (23.7)	1649 (28.7)	1878 (32.5)	2019 (35.0)	
high school	1066 (18.4)	1230 (21.4)	1321 (22.9)	1410 (24.4)	
>high school	3362 (57.9)	2875 (50.0)	2571 (44.6)	2342 (40.6)	
Sex (%)					<0.001
Male	3449 (59.4)	2979 (51.8)	2511 (43.5)	2266 (39.3)	
Female	2355 (40.6)	2775 (48.2)	3259 (56.5)	3505 (60.7)	
Smoker (%)					<0.001
Yes	4186 (72.1)	4340 (75.4)	4436 (76.9)	4538 (78.6)	
No	1618 (27.9)	1414 (24.6)	1334 (23.1)	1233 (21.4)	
FBG (mg/dl)	105.50 ± 32.89	106.59 ± 33.95	107.70 ± 36.35	107.24 ± 35.59	0.004
TC (mmol/l)	5.05 ± 1.09	5.05 ± 1.09	5.05 ± 1.13	4.97 ± 1.11	<0.001
Race (%)					<0.001
Mexican American	1183 (20.4)	1103 (19.2)	1078 (18.7)	929 (16.1)	
Other Hispanic	419 (7.2)	486 (8.4)	465 (8.1)	390 (6.8)	
Non-Hispanic White	2760 (47.6)	2588 (45.0)	2564 (44.4)	2291 (39.7)	
Non-Hispanic Black	821 (14.1)	1015 (17.6)	1237 (21.4)	1638 (28.4)	
Other Races	621 (10.7)	562 (9.8)	426 (7.4)	523 (9.1)	
eGFR (%)					<0.001
CKD stage G1	3962 (68.3)	3967 (68.9)	3839 (66.5)	3827 (66.3)	
CKD stage G2	1585 (27.3)	1468 (25.5)	1522 (26.4)	1468 (25.4)	
CKD stage G3	244 (4.2)	280 (4.9)	370 (6.4)	421 (7.3)	
CKD stage G4	10 (0.2)	32 (0.6)	28 (0.5)	35 (0.6)	
CKD stage G5	3 (0.1)	7 (0.1)	11 (0.2)	20 (0.3)	

SBP, systolic blood pressure; eGFR, estimation of glomerular filtration rate; FBG, fasting blood glucose; BMI, body mass index; CKD, chronic kidney disease; HDL-C, low-density lipoprotein cholesterol; TC, total cholesterol; TG, total triglycerides; LDL-C, low-density lipoprotein cholesterol.

### 3.2 Associations of participants’ DII index, TyG index, WTI index and BMI index with Kaplan-Meier survival curves of CKD patients

**Figure 2:**
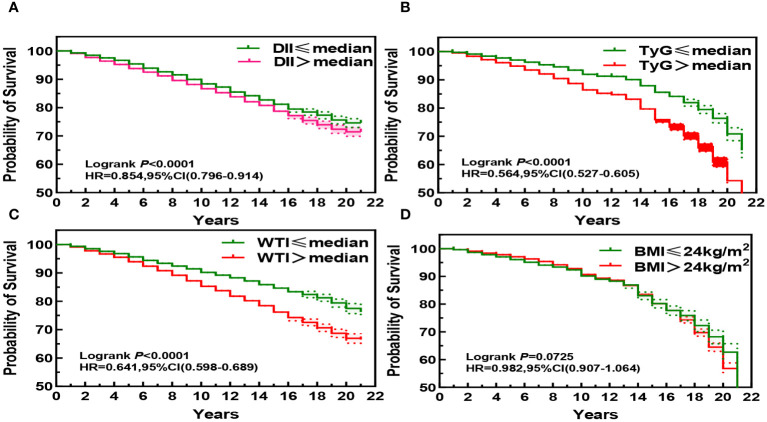
Kaplan-Meier Survival Curves for DII **(A)**, TyG **(B)**, WTI **(C)** and BMI **(D)** in chronic kidney disease.

The Kaplan-Meier curve shows that the mortality rate of CKD patients significantly increases (P< 0.001) when DII index, WTI index, and TyG index reach or exceed the median, indicating that the higher these three indices are, the higher the risk of death for CKD patients. Specifically, higher DII index was associated with increased risk of mortality in CKD patients (HR, 0.854; 95%CI, 0.796–0.914, **Figure 2A**), while higher WTI index was also associated with increased risk of mortality in CKD patients (HR, 0.641; 95%CI, 0.598–0.689, **Figure 2B**), and higher TyG index was associated with increased risk of mortality in CKD patients (HR, 0.564; 95%CI, 0.527–0.605, **Figure 2C**). However, BMI index did not show statistical significance on the survival curve of CKD patients (P > 0.05, **Figure 2D**).

### 3.3 Univariate and multivariate COX regression risk models of DII index, WTI index and TyG index with the mortality risk of CKD patients

**Figure 3:**
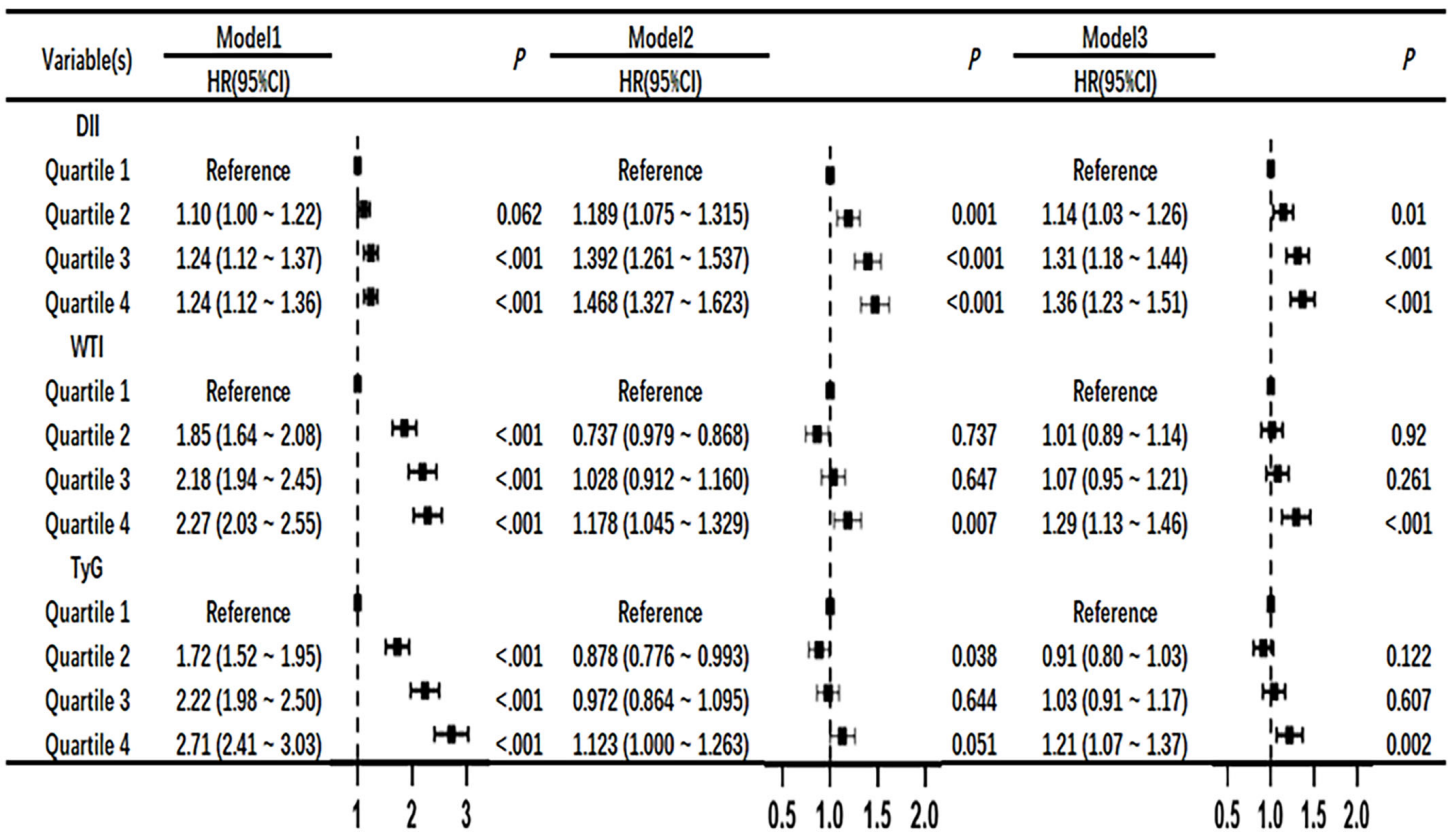
Association of DII, WTI and TyG with chronic kidney disease in Cox proportional hazard models. Model 1,unadjsted. Model 2, adjusted for age, sex and BMI. Model 3,further adjusted for variables in model 2 plus smoking status, race, education level, TC, systolic blood pressure.

**Figure 4:**
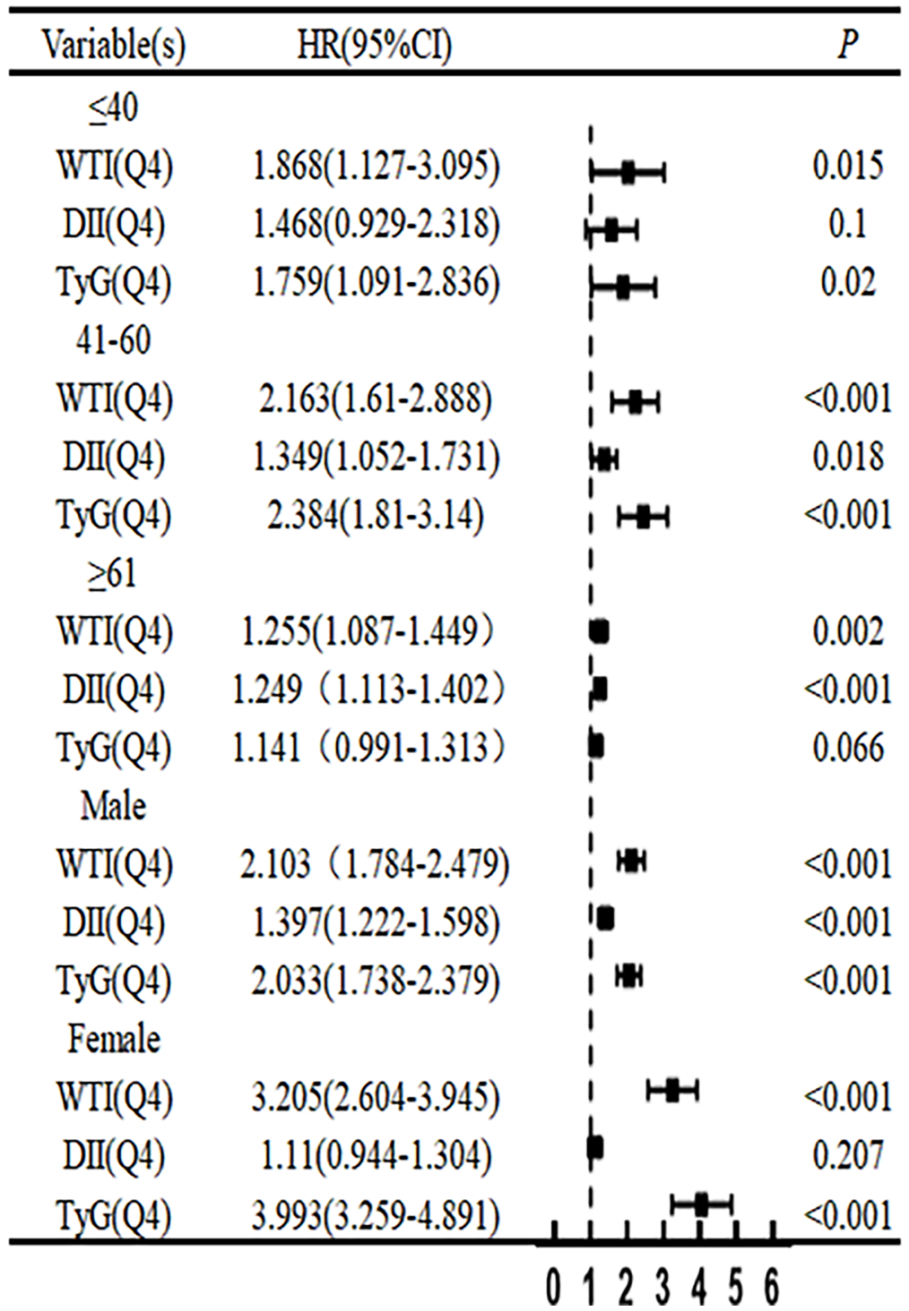
Adjusted HRs (95% CIs) for risk of chronic kidney disease according to sex- and age-specific WTI, DII and TyG index.

After adjusting for the COX risk model in **Figure 3**, the risk ratio (HR) of death from CKD significantly increased when the DII index was greater than or equal to the median, and the P values were statistically significant (P<0.05). The WTI index showed a significant statistical significance in the fourth quartile for the risk of death from CKD (HR, 1.29; 95% CI 1.13–1.46; P<0.001), and the TyG index also showed a significant statistical significance in the fourth quartile for the risk of death from CKD (HR, 1.21; 95% CI 1.07–1.37; P=0.002). The eGFR is greatly influenced by age and gender, as shown in **Figure 4** where age and gender are stratified. For individuals aged 40 or younger, after adjusting for confounding factors, the fourth quartile of TyG index (P=0.02) and WTI index (P=0.015) were statistically significant in relation to the risk of death in CKD patients. For those aged 41 to 60, after adjusting for confounding factors, the fourth quartile of WTI index (P<0.001), DII index (P=0.018) and TyG index (P<0.001) were statistically significant in relation to the risk of death in CKD patients. Individuals aged 61 years and older, after adjusting for confounding factors in the fourth quartile, had a statistically significant association between WTI index (P<0.001), DII index (P=0.018) and TyG index (P<0.001) with the risk of death in CKD patients. In males, after adjusting for confounding factors in the fourth quartile, there was a statistically significant association between WTI index (P<0.001), DII index (P<0.001), and TyG index (P<0.001) with the risk of death in CKD patients. In females, after adjusting for confounding factors in the fourth quartile, there was a statistically significant association between WTI index (P<0.001) and TyG index (P<0.001) with the risk of death in CKD patients.

### 3.4 The correlation between DII index, TyG index and WTI index with the risk of death in CKD patients

**Figure 5:**
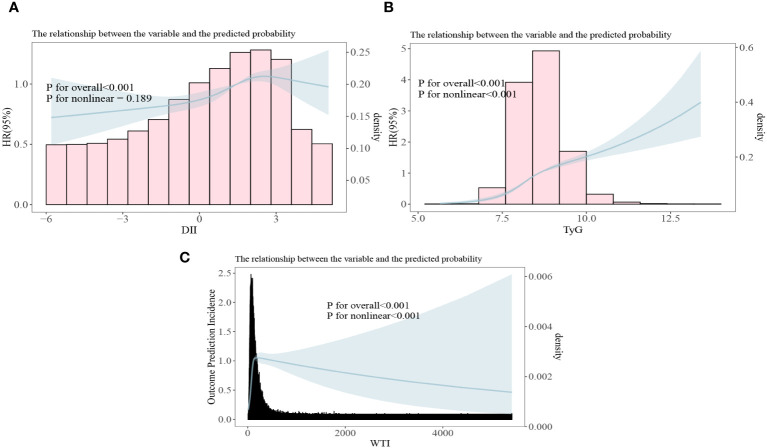
All-cause mortality risk ratios of chronic kidney disease to three indices [**(A)** DII, Dietary Inflammation Index; **(B)** TyG, Triglyceride and Glucose Index; **(C)** WTI, Waist-Triglyceride Index].

In the restrictive cubic spline analysis, the DII index showed a linear relationship with the risk ratio of mortality in participants with CKD (non-linear p=0.189; **Figure 5A**). The TyG index exhibited an increasing relationship with the risk ratio of mortality in participants with CKD (non-linear p<0.001; **Figure 5B**). The WTI index demonstrated a hook-shaped relationship with the risk ratio of mortality in participants with CKD, initially rising and then declining (non-linear p<0.001; **Figure 5C**).

### 3.5 Equations

Calculation formula for estimating bilateral glomerular filtration rate (eGFR) based on an all-age spectrum correction equation for creatinine in 2021 (22). For females, the value of C was taken as 1.012. When serum creatinine was less than or equal to 0.7 mg/dl, the value of A was 0.7 and the value of B was -0.241. When serum creatinine is greater than 0.7mg/dl, the A value is 0.7 and the B value is taken as -1.2. For males, the C value is taken as 1. When serum creatinine is less than or equal to 0.9 mg/dl, the A value is 0.9 and the B value is taken as -0.302. When serum creatinine is greater than 0.9mg/dl, A value is 0.9 and B value is taken as -1.2.


$$\text{eGFR}\;=\;142\;*{\left(\frac{Scr}{A}\right)}^{B}*\;{\left(0.9938\right)}^{Age}*\;C$$

## 4 Discussion

Based on a cross-sectional analysis of 23,099 U.S. adults, the DII index had the most significant value for predicting chronic kidney disease in adults compared to the TyG index and the WTI index. This result was not obvious in univariate analysis, but showed significant difference after adjusting for age, sex and race.

In earlier studies, a retrospective study discussed the correlation between TyG index and CKD in American adults (20, 21), and concluded that CKD is more likely to occur when TyG index is high (23, 24). The TyG index also has the same trend in predicting the occurrence of chronic kidney disease in Japanese population (25). Therefore, TyG index may become one of the risk predictors of chronic kidney disease (26). The DII index aims to provide a quantitative means of assessing the relationship between diet and health outcomes ranging from blood concentrations of inflammatory cytokines to chronic disease (27). DII index is mainly used to evaluate the influence of diet on inflammatory response and help prevent and treat inflammation-related diseases, such as cardiovascular disease, diabetes, tumor, etc. (16, 28, 29). However, there are few studies on DII index predicting the occurrence of chronic disease CKD in American population. WTI index was used by Korean researchers to investigate the waist combined with triglycerides to predict the occurrence of cardiovascular disease and metabolic syndrome (13, 30). To our knowledge, no studies have explored the correlation between the WTI index and CKD. Compared with previous studies, our study has some advantages. First, we verified the TyG index and DII index obtained in previous studies to predict the risk of CKD in American adults and introduced a new WTI index to predict the occurrence of CKD. It is worth noting that our study can take TyG index as a reference, and through sensitivity analysis, it is found that the risk of CKD occurrence is increasing with the increase of DII index, which is consistent with previous studies on the correlation between DII index and CKD index (31). This study further concluded that DII index predicts the occurrence of CKD better than other indexes. In addition, the high WTI index is stronger than the TyG index in predicting CKD. In the nonlinear relationship between TyG index and CKD, the death risk of CKD increased with the increase of TyG index. In the non-linear relationship between WTI index and CKD, the risk of death in CKD decreased slowly with the increase of WTI index, which may be related to the differences of sample size, eGFR calculation method, population, race and region.

Many studies have pointed out that high DII index is correlated with chronic diseases such as cardiovascular disease, diabetes mellitus and sarcopenia (16, 32–34), while there are few studies on the relationship between DII index and CKD. DII index is a comprehensive score reflecting pro-inflammatory diet and anti-inflammatory diet. A high DII index means that the diet contains a large amount of inflammatory factors and a small amount of anti-inflammatory nutrients (35), and excessive intake of pro-inflammatory diet may have adverse effects on health (36). Intake of a pro-inflammatory diet (higher DII score) was associated with elevated levels of various inflammatory markers: TNF-α, highly sensitive C-reactive protein (hsCRP), IL-1, 2, 6, IFN-γ and vascular cell adhesion molecules (37–39), moreover, adding one DII scoring unit in men increased hsCRP by 9% (95%CI 0.03–0.14) and IL-6 by 6% (95%CI 0.02–0.11) in patients with first-time myocardial infarction (40). Therefore, the mechanism by which it affects CKD may be that a high DII diet leads to the accumulation of inflammatory factors in the body, increasing the level of oxidative stress, which damages cells and tissues (41) thus leading to an increased risk of CKD in people. Recent studies have shown that higher intake of specific antioxidants and anti-inflammatory dietary components can affect telomere length and thus delay aging, for example, increased intake of omega-3 fatty acids and vitamin C is associated with longer telomere (42, 43), and HDL-C concentration is positively correlated when telomere length is less than 1.25 (44). However, how DII index affects kidney function in CKD patients in which pathways at the cellular level needs to be confirmed by further experimental studies. The advantage of the DII index compared to the other two indexes is that the clinician can conduct a detailed and comprehensive analysis of the potential factors for reducing the risk of developing CKD through the intake of a precise anti-inflammatory diet and a pro-inflammatory diet.

Our study possesses several notable strengths including a substantial sample size and meticulous adjustment for pertinent confounding variables that have enhanced both reliability and representativeness of our findings. The Dietary Inflammatory Index (DII) holds promise as a comprehensive therapeutic tool encompassing 45 different diets impacting individuals’ health; therefore, clinicians can potentially mitigate chronic kidney disease (CKD) incidence by modifying dietary components among those at risk for CKD. Nonetheless, there are limitations within our study framework; due to its cross-sectional nature, establishing causality between DII scores and CKD was precluded. While we accounted for numerous critical factors, complete exclusion of potential effects from unmeasured confounders was not feasible. Furthermore, given that our survey focused on the U.S. population using a cross-sectional approach, generalizing our results to other demographic or ethnic cohorts may be limited.

## 5 Conclusion

Compared with the TyG index and WTI index, the DII index is more effective in predicting CKD in U.S. adults. People with high DII index, TyG index or WTI index should pay close attention to their potential risk to kidney function. Nevertheless, more comprehensive prospective studies are needed to confirm and validate these results.

## Data availability statement

The original contributions presented in the study are included in the article/supplementary material. Further inquiries can be directed to the corresponding author.

## Ethics statement

The studies involving humans were approved by National Center for Health Statistics Research Ethics Review Board. The studies were conducted in accordance with the local legislation and institutional requirements. The participants provided their written informed consent to participate in this study.

## Author contributions

ZL: Data curation, Writing – original draft, Formal analysis, Writing – review & editing. ZX: Writing – review & editing. CX: Writing – review & editing. HX: Writing – review & editing.

## References

[1] Obrador GT Pereira BJ Kausz AT. Chronic kidney disease in the United States: an underrecognized problem. Semin Nephrol. (2002) 22:441–8. doi: 10.1053/snep.2002.2002.35962 12430088

[2] Thomas C Thomas L. Renal failure–measuring the glomerular filtration rate. Dtsch Arztebl Int. (2009) 106:849–54. doi: 10.3238/arztebl.2009.0849 PMC2803612 20062583

[3] Balderas-Vargas NA Legorreta-Soberanis J Paredes-Solis S Flores-Moreno M Santos F Andersson N. Occult renal failure and associated factors in patients with chronic conditions. Gac Med Mex. (2020) 156:11–6. doi: 10.24875/GMM.19005292 32026875

[4] Banerjee T Liu Y Crews DC. Dietary patterns and CKD progression. Blood Purif. (2016) 41:117–22. doi: 10.1159/000441072 26765365

[5] Molina P Gavela E Vizcaino B Huarte E Carrero JJ. Optimizing diet to slow CKD progression. Front Med (Lausanne). (2021) 8:654250. doi: 10.3389/fmed.2021.654250 34249961 PMC8267004

[6] Jankowski J Floege J Fliser D Bohm M Marx N. Cardiovascular disease in chronic kidney disease: pathophysiological insights and therapeutic options. Circulation. (2021) 143:1157–72. doi: 10.1161/CIRCULATIONAHA.120.050686 PMC7969169 33720773

[7] Ku E Lee BJ Wei J Weir MR. Hypertension in CKD: core curriculum 2019. Am J Kidney Dis. (2019) 74:120–31. doi: 10.1053/j.ajkd.2018.12.044 30898362

[8] Zelnick LR Weiss NS Kestenbaum BR Robinson-Cohen C Heagerty PJ Tuttle K. Diabetes and CKD in the United States population, 2009-2014. Clin J Am Soc Nephrol. (2017) 12:1984–90. doi: 10.2215/CJN.03700417 PMC5718269 29054846

[9] Jiang Z Wang Y Zhao X Cui H Han M Ren X. Obesity and chronic kidney disease. Am J Physiol Endocrinol Metab. (2023) 324:E24–41. doi: 10.1152/ajpendo.00179.2022 36383637

[10] Khan SH Sobia F Niazi NK Manzoor SM Fazal N Ahmad F. Metabolic clustering of risk factors: evaluation of Triglyceride-glucose index (TyG index) for evaluation of insulin resistance. Diabetol Metab Syndr. (2018) 10:74. doi: 10.1186/s13098-018-0376-8 30323862 PMC6173832

[11] Ramdas Nayak VK Satheesh P Shenoy MT Kalra S. Triglyceride Glucose (TyG) Index: A surrogate biomarker of insulin resistance. J Pak Med Assoc. (2022) 72:986–8. doi: 10.47391/JPMA.22-63 35713073

[12] Li H Chen W Lin X Chen W Xie T Chen K. Influence of renal function on the ability of TyG Index to predict all-cause mortality. Lipids Health Dis. (2023) 22:193. doi: 10.1186/s12944-023-01958-1 37951945 PMC10638822

[13] Liu PJ Lou HP Zhu YN. Screening for metabolic syndrome using an integrated continuous index consisting of waist circumference and triglyceride: A preliminary cross-sectional study. Diabetes Metab Syndr Obes. (2020) 13:2899–907. doi: 10.2147/DMSO.S259770 PMC7443454 32884316

[14] Chung KH Choi YH Cho IR Son BK Ryu JK Kim YT. Hypertriglyceridaemic waist phenotype and waist circumference triglyceride index are associated with higher incidence of acute pancreatitis: a nationwide population-based retrospective cohort study. BMJ Open. (2023) 13:e071213. doi: 10.1136/bmjopen-2022-071213 PMC10465893 37643853

[15] Shu Y Wu X Wang J Ma X Li H Xiang Y. Associations of dietary inflammatory index with prediabetes and insulin resistance. Front Endocrinol (Lausanne). (2022) 13:820932. doi: 10.3389/fendo.2022.820932 35250879 PMC8892213

[16] King DE Xiang J. The dietary inflammatory index is associated with diabetes severity. J Am Board Fam Med. (2019) 32:801–6. doi: 10.3122/jabfm.2019.06.190092 PMC6986800 31704748

[17] Huang Y Zeng M Zhang L Shi J Yang Y Liu F. Dietary inflammatory potential is associated with sarcopenia among chronic kidney disease population. Front Nutr. (2022) 9:856726. doi: 10.3389/fnut.2022.856726 35634405 PMC9131018

[18] Di X Xiang L Jian Z Xia Z Luo D. Association between urinary phthalate metabolites and nephrolithiasis in adults: A cross-sectional analysis with NHANES 2007-2018. Chemosphere. (2023) 337:139436. doi: 10.1016/j.chemosphere.2023.139436 37422213

[19] Li Y Di X Liu M Wei J Li T Liao B. Association between daily sitting time and kidney stones based on the National Health and Nutrition Examination Survey (NHANES) 2007-2016: A cross-sectional study. Int J Surg. (2024). doi: 10.1097/JS9.0000000000001560 PMC11325893 38768465

[20] Liu N Liu C Qu Z Tan J. Association between the triglyceride-glucose index and chronic kidney disease in adults. Int Urol Nephrol. (2023) 55:1279–89. doi: 10.1007/s11255-022-03433-9 36472799

[21] Li X Wang L Zhou H Xu H. Association between triglyceride-glucose index and chronic kidney disease: results from NHANES 1999-2020. Int Urol Nephrol. (2024). doi: 10.1007/s11255-024-04103-8 PMC11464617 38856937

[22] Pottel H Bjork J Courbebaisse M Couzi L Ebert N Eriksen BO. Development and validation of a modified full age spectrum creatinine-based equation to estimate glomerular filtration rate: A cross-sectional analysis of pooled data. Ann Intern Med. (2021) 174:183–91. doi: 10.7326/M20-4366 33166224

[23] Ren X Jiang M Han L Zheng X. Association between triglyceride-glucose index and chronic kidney disease: A cohort study and meta-analysis. Nutr Metab Cardiovasc Dis. (2023) 33:1121–8. doi: 10.1016/j.numecd.2023.03.026 37088649

[24] Kunutsor SK Seidu S Kurl S Laukkanen JA. Baseline and usual triglyceride-glucose index and the risk of chronic kidney disease: a prospective cohort study. Geroscience. (2024) 46:3035–46. doi: 10.1007/s11357-023-01044-5 PMC11009217 38180700

[25] Sakoda T Akasaki Y Sasaki Y Kawasoe S Kubozono T Ikeda Y. Triglyceride-glucose index predicts future chronic kidney disease development in all populations, including normotensive and isolated diastolic hypertension. Hypertens Res. (2024) 47:149–56. doi: 10.1038/s41440-023-01507-4 37989912

[26] Okamura T Hashimoto Y Hamaguchi M Obora A Kojima T Fukui M. Triglyceride-glucose index is a predictor of incident chronic kidney disease: a population-based longitudinal study. Clin Exp Nephrol. (2019) 23:948–55. doi: 10.1007/s10157-019-01729-2 30955189

[27] Hebert JR Shivappa N Wirth MD Hussey JR Hurley TG. Perspective: the dietary inflammatory index (DII)-lessons learned, improvements made, and future directions. Adv Nutr. (2019) 10:185–95. doi: 10.1093/advances/nmy071 PMC6416047 30615051

[28] Ji M Hong X Chen M Chen T Wang J Zhang N. Dietary inflammatory index and cardiovascular risk and mortality: A meta-analysis of cohort studies. Med (Baltimore). (2020) 99:e20303. doi: 10.1097/MD.0000000000020303 PMC7253850 32443378

[29] Zhang C Wang W Zhang D. Association between dietary inflammation index and the risk of colorectal cancer: A meta-analysis. Nutr Cancer. (2018) 70:14–22. doi: 10.1080/01635581.2017.1374418 29087221

[30] Yang RF Liu XY Lin Z Zhang G. Correlation study on waist circumference-triglyceride (WT) index and coronary artery scores in patients with coronary heart disease. Eur Rev Med Pharmacol Sci. (2015) 19:113–8. 25635983

[31] Mazidi M Shivappa N Wirth MD Hebert JR Kengne AP. Greater Dietary Inflammatory Index score is associated with higher likelihood of chronic kidney disease. Br J Nutr. (2018) 120:204–9. doi: 10.1017/S0007114518001071 29947319

[32] Garcia-Arellano A Ramallal R Ruiz-Canela M Salas-Salvado J Corella D Shivappa N. Dietary inflammatory index and incidence of cardiovascular disease in the PREDIMED study. Nutrients. (2015) 7:4124–38. doi: 10.3390/nu7064124 PMC4488776 26035241

[33] Hariharan R Odjidja EN Scott D Shivappa N Hebert JR Hodge A. The dietary inflammatory index, obesity, type 2 diabetes, and cardiovascular risk factors and diseases. Obes Rev. (2022) 23:e13349. doi: 10.1111/obr.13349 34708499

[34] HassUHerpichCKochlikBWeberDGruneTNormanK. Dietary inflammatory index and cross-sectional associations with inflammation, muscle mass and function in healthy old adults. J Nutr Health Aging. (2022) 26:346–51. doi: 10.1007/s12603-022-1753-4 35450990

[35] Wang YB Shivappa N Hebert JR Page AJ Gill TK Melaku YA. Association between dietary inflammatory index, dietary patterns, plant-based dietary index and the risk of obesity. Nutrients. (2021) 13. doi: 10.3390/nu13051536 PMC8147427 34063221

[36] Phillips CM Shivappa N Hebert JR Perry IJ. Dietary inflammatory index and biomarkers of lipoprotein metabolism, inflammation and glucose homeostasis in adults. Nutrients. (2018) 10. doi: 10.3390/nu10081033 PMC6115860 30096775

[37] Shivappa N Hebert JR Rietzschel ER De Buyzere ML Langlois M Debruyne E. Associations between dietary inflammatory index and inflammatory markers in the Asklepios Study. Br J Nutr. (2015) 113:665–71. doi: 10.1017/S000711451400395X PMC4355619 25639781

[38] Shivappa N Hebert JR Marcos A Diaz LE Gomez S Nova E. Association between dietary inflammatory index and inflammatory markers in the HELENA study. Mol Nutr Food Res. (2017) 61. doi: 10.1002/mnfr.201600707 PMC5517083 27981781

[39] Shin D Lee KW Brann L Shivappa N Hebert JR. Dietary inflammatory index is positively associated with serum high-sensitivity C-reactive protein in a Korean adult population. Nutrition. (2019) 63-64:155–61. doi: 10.1016/j.nut.2018.11.016 30999247

[40] Boden S Wennberg M Van Guelpen B Johansson I Lindahl B Andersson J. Dietary inflammatory index and risk of first myocardial infarction; a prospective population-based study. Nutr J. (2017) 16:21. doi: 10.1186/s12937-017-0243-8 28376792 PMC5379659

[41] Duan Y Zeng L Zheng C Song B Li F Kong X. Inflammatory links between high fat diets and diseases. Front Immunol. (2018) 9:2649. doi: 10.3389/fimmu.2018.02649 30483273 PMC6243058

[42] Farzaneh-Far R Lin J Epel ES Harris WS Blackburn EH Whooley MA. Association of marine omega-3 fatty acid levels with telomeric aging in patients with coronary heart disease. JAMA. (2010) 303:250–7. doi: 10.1001/jama.2009.2008 PMC2819264 20085953

[43] Cai Y Zhong YD Zhang H Lu PL Liang YY Hu B. Association between dietary vitamin C and telomere length: A cross-sectional study. Front Nutr. (2023) 10:1025936. doi: 10.3389/fnut.2023.1025936 36776610 PMC9908946

[44] Chen YF Zhou KW Yang GZ Chen C. Association between lipoproteins and telomere length in US adults: data from the NHANES 1999-2002. Lipids Health Dis. (2019) 18:80. doi: 10.1186/s12944-019-1030-7 30935416 PMC6444542

